# Copper Monitoring in Vineyard Soils of Central Italy Subjected to Three Antifungal Treatments, and Effects of Sub-Lethal Copper Doses on the Earthworm *Eisenia fetida*

**DOI:** 10.3390/toxics10060310

**Published:** 2022-06-08

**Authors:** Arianna De Bernardi, Enrica Marini, Cristiano Casucci, Luca Tiano, Fabio Marcheggiani, Costantino Vischetti

**Affiliations:** 1 Department of Agricultural, Food and Environmental Sciences, Polytechnic University of Marche, 60131 Ancona, Italy; a.debernardi@pm.univpm.it (A.D.B.); enrica.marini@staff.univpm.it (E.M.); c.casucci@univpm.it (C.C.); 2 Department of Life and Environmental Sciences, Polytechnic University of Marche, 60131 Ancona, Italy; l.tiano@univpm.it (L.T.); f.marcheggiani@pm.univpm.it (F.M.)

**Keywords:** copper fungicide, soil contamination, ecotoxicology, earthworms, *Eisenia fetida*, comet assay, vineyard

## Abstract

The extensive employment of copper-based fungicides has increased copper concentration in vineyard soils. The present study’s objectives were to monitor copper concentration in two vineyard soils during two cropping seasons and study the ecotoxicological effects on the earthworm *Eisenia fetida*. Total, soluble, and bioavailable copper fractions were measured at the end of two cropping seasons and different depths in two vineyards of central Italy, characterised by three anticryptogamic control methods: copper compounds, chitosan, and combined treatments of them. A laboratory experiment to assess the effects on *Eisenia fetida* was conducted with soil samples collected in the vineyards with a mean copper concentration of 60 mg/kg and two higher concentrations of 90 and 150 mg/kg. Results showed low levels of total copper concentration in the first 20 cm of soils, regardless of antifungal treatment, highlighting prudent management of the vineyards under study, but the soluble fractions showed a significant increase in all samples during the two cropping seasons. At the dose of 150 mg/kg, earthworms suffer during the first two days, showing weight loss and DNA damage, but they are able to recover until day 28, showing no permanent harm at this copper concentration in soil.

## 1. Introduction

Copper (Cu) is an essential micronutrient, but when in excess, it can be harmful to biological systems. Due to anthropogenic activities, the concentration of Cu has increased in the environment and can affect human health, considering the possibility of a dietary intake associated with contaminated crops [1,2]. If ingested in large quantities, Cu can cause hepatic and neurological diseases [3]. 

One of the major anthropogenic sources of copper contamination in soils is the long-term use of Cu-based compounds in agrochemistry which has contributed to a significant increase in soil Cu concentrations over recent decades [4,5,6]. Azimi et al. estimate an annual copper deposition for agricultural inputs that varies between 100 and 800 g Cu/km^2^ depending on fertiliser and type of crop [7].

A recent large-scale study showed the highest Cu concentrations in vineyard soils, among other land uses, with a mean concentration of 49.3 mg/kg, which is three times higher than the average general concentration in European soils; additionally, copper contamination occurs mainly in wet areas due to frequent fungicide treatments. Copper values reported by this survey show high variability between samples, of which, a considerable percentage (14.6%) exceeds 100 mg/kg [8].

Many worrying cases regarding copper in vineyard soils were detected at the European level [9]. For instance, concentrations around 200–400 mg/kg were found in French soils [10,11,12], while in Spain, values ranging between 500 and 600 mg/kg were measured [13,14]; furthermore, in some Croatian vineyards, Cu reached 700 mg/kg [15].

In Italy, several studies and regional surveys report highly variable concentrations of copper among vineyards, ranging from 30 to over 300 mg/kg, so there are cases in which copper exceeds the national law threshold indicated for land for agricultural use of 200 mg/kg [16,17,18,19,20].

The biological nature of metal bioavailability requires biological assays to understand its mechanism of toxicity [21]. Among soil organisms, earthworms are more susceptible to metal pollution than many other soil invertebrates and could be used as biological tools in ecotoxicological studies [22,23,24,25]. Several earthworm toxicity assays relative to different thresholds can be studied to evaluate the potential adverse effects of xenobiotics on the environment [26]. While chemical analytical methods alone are not adequate for a comprehensive quantification of metal bioavailability and Cu speciation in soils, biological parameters can provide a useful complementary approach to the evaluation of Cu bioavailability in soil [27].

As copper-based compounds used in agriculture meet the criteria of persistence and toxicity, the Commission Implementing Regulation 2015/408 [28] included these substances (copper hydroxide, copper oxychloride, Bordeaux mixture, tribasic copper sulphate, and copper oxide) in the list of chemical candidates for substitution. Currently, the European Commission concluded that further ecotoxicological studies, specifically on soil organisms, are needed for updated risk assessment [29]. 

Karimi et al. [30] reported that the primary consumers of copper-based fungicides, organic viticulture, presented better soil biological quality than conventional viticulture, but the relative contribution of each viticultural practice could not be established, and no data on the effect of Cu application was provided. A No Observed Adverse Effect concentration (NOAEC) of 4 kg Cu/ha/year on average was reported from a study considering the transient effects on abundance and biomass of earthworms [31]. 

From an exhaustive meta-analysis on Cu ecotoxicology relative to 19 articles out of 300 papers relevant to copper and soil biological quality performed by Karimi et al. [32], it was assessed that Cu accumulated in the soil over the years started to be deleterious for earthworm’s biomass at concentrations above 200 kg Cu/ha/year. Moreover, literature analysis shows a substantial gap in the relation to ecotoxicological effects at doses below 4 kg Cu/ha/year chronic contamination, in particular, in relation to the long-term effects on soil biodiversity [32]. This data highlights the need for studies that address the environmental risk linked to the historical use of copper in European vineyard soils.

To define the xenobiotics’ potential risk to the environment, in addition to ecotoxicological studies that take into account parameters such as biomass or mortality, the use of genotoxic biomarkers is recognised [26,33]. In this context, the comet assay is considered a valid and sensitive method for assessing early damage to the DNA of organisms such as earthworms, regarded as sentinels of the state of health of the soil [33,34,35,36,37].

Moreover, at the European level, research has been promoted on alternative products to supplement or substitute cupric fungicides, focusing on natural compounds registered as plant protection products (PPP) or plant biostimulants [38]. Among the substitutes for copper in the antifungal fight in the vineyard, growing interest is observed for chitosan as it is a biocompatible, biodegradable, and non-toxic polymer molecule [39]. 

The main objective of the present study was to verify the effects of copper concentrations in the soil due to the application of three different antifungal control strategies: (i) cupric compounds, (ii) chitosan, and (iii) alternation of cupric compounds and chitosan.

The three copper fractions (total, bioavailable, and soluble) were monitored in two commercial organic vineyards conducting experiments for two years to test the long-term effects of natural alternative treatments to copper.

It has been hypothesised that the careful management of treatments, following national guidelines, and using new generation antifungals as partial or total substitutes for cupric compounds may not cause concern in the accumulation of this element in the soil.

Furthermore, a second objective was to better assess the impact of copper, at three sub-lethal doses, on the soil ecosystem by conducting an ecotoxicological test with earthworms on mesocosms set up with natural soil and which provided early damage detection through the comet assay technique.

## 2. Materials and Methods

### 2.1. Monitoring Campaign

#### 2.1.1. Study Areas

This study was carried out in two pilot vineyards in the Ancona province (Marche Region, Central Italy), about 37 km from each other. Specifically, one vineyard (V) is in the Varano hamlet of Ancona (centroid coordinates 43°33′11.42″ N; 13°32′10.85″ E), while the other (C) is located in the Castelplanio municipality (centroid coordinates 43°30′16.03″ N; 13° 4′54.31″ E). As stated in the only complete Italian map that takes into account the Worldwide Bioclimatic Classification System according to Rivas-Martínez [40], both vineyards fall into the macro-bioclimate Temperate and the Sub-Mediterranean bioclimatic variant [41]. The following bioclimatic units (in top-down hierarchical order) are bioclimate Oceanic Temperate, thermotype Mesotemperate (Lower and Upper, respectively, for V and C vineyard), and ombrotype Subhumid (Lower and Upper, respectively, for V and C vineyard).

According to what is indicated in Italy’s soil map and the Typological Units database [42], the types of soil can be catalogued in Haplic Calcisol and Calcaric Cambisol, respectively, for Castelplanio and Varano.

In both organic vineyards, experiments are underway on different antifungal treatments. For this reason, each vineyard is interested in several treatments and each antifungal treatment is conducted in groups of three consecutive rows. The length of the rows in the two vineyards is not regular; in the Varano vineyard, the average length of a row is 150 m, while in the case of Castelplanio it is 100 m. The distance between the rows is fixed at 2.2 m and 2.5 m for the Varano and Castelplanio vineyards, respectively.

#### 2.1.2. Sampling

Two monitoring campaigns were carried out at the end of the harvest (October 2020/October 2021) in the two vineyards C and V.

In both vineyards, the sampling regarded the rows in which the following antifungal treatments are applied:

(A) Copper treatments (use of copper-based products for the entire growing season);
(B) Alternate treatments (use of copper in the first part of the season and chitosan at 0.5% of p.a. after flowering);
(C) Chitosan treatments (use of 0.5% p.a. chitosan for the entire growing season).

In (A), an average of 2.5 kg/ha of copper metal (Cu^2+^) was used, mainly distributed in the form of copper sulphate tribasic, neutralised copper sulphate, copper hydroxide, and cuprous oxide, while for (B), the use of an average of 1 kg/ha of Cu metal distributed with the same products was estimated. The number of Cu treatments is summarised in Table 1, for the two years 2020 and 2021.

**Figure 1:**
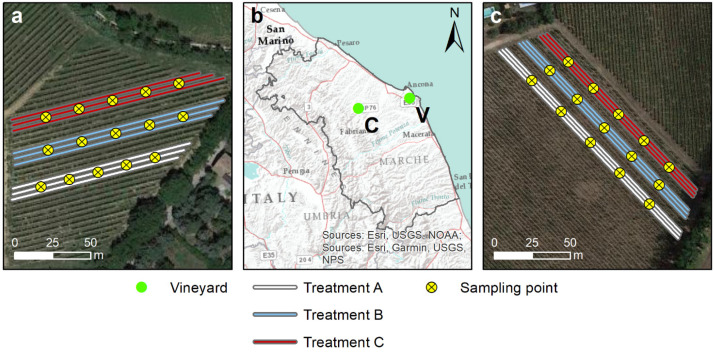
Sampling map in the two vineyards under study. (**a**) Sampling in Castelplanio vineyard, (**b**) Location of the two vineyards Castelplanio (C) and Varano (V) in the Marche region, and (**c**) Sampling in Varano vineyard. Treatments refer to the antifungal strategies applied on the rows: (A) Cu treatments, (B) Alternating treatments with Cu and chitosan, and (C) Chitosan treatments.

For each plot consisting of three contiguous rows and relating to one of the three theses previously indicated (A, B, and C), the central row was selected (to avoid drift contamination), and five equidistant sampling points were spaced along the entire length of the row as reported in Figure 1a,c. In each of these five points, two soil samples were taken at the two depths of 0–20 cm (Z) and 20–40 cm (Q).

This sampling design was used in both vineyards in 2020 and was repeated in the same way in 2021. 

Each sample was air-dried, ground manually, and sieved at 2 mm to obtain about 3 kg of fine earth for each sample.

To obtain three homogeneous samples for each depth (Z and Q) and thesis (A, B, and C), the same soil portions of each of the five sampling points were suitably mixed for a total of 12 macro-samples (3 theses × 2 vineyards × 2 depths), each analysed for physico-chemical characteristics (Table 2).

To get a picture of the two vineyards’ climatic situation and the two years of sampling, data relating to the rainfall regimes and median temperatures measured in weather stations closest to the two vineyards from the Marche Civil Protection database [44] were analysed and processed. For the Varano vineyard (V), station 613-Baraccola (sensor code 2854) was selected, while for the Castelplanio (C) vineyard, station 506-Moie (sensor code 3021) was used.

**Figure 2:**
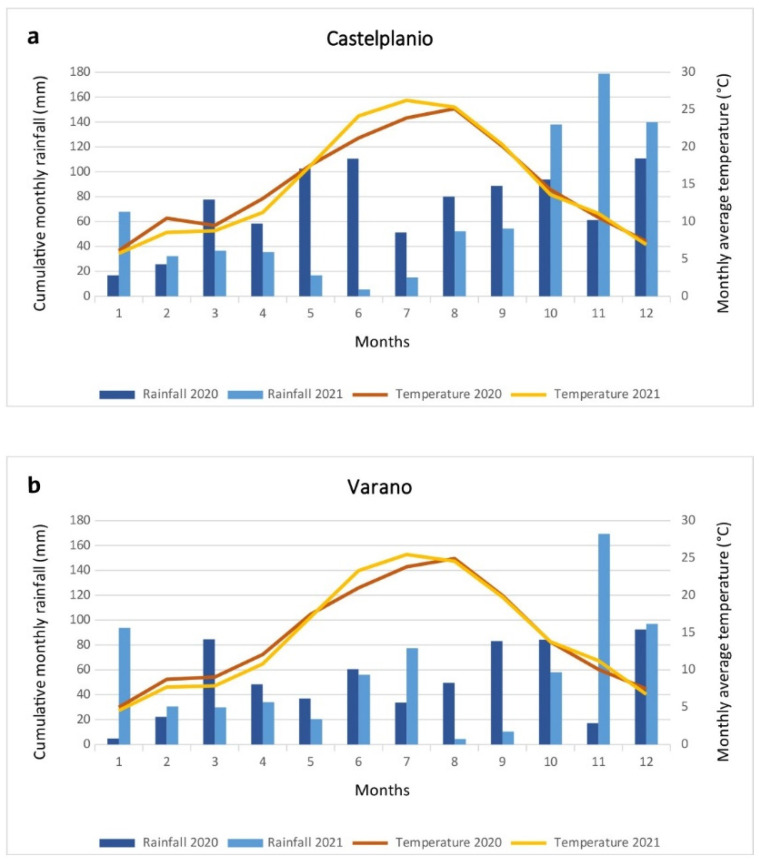
Climatic conditions in the two vineyards under study in 2020 and 2021. (**a**) Castelplanio vineyard, (**b**) Varano vineyard.

It can be observed from Figure 2 that the temperature trend was very similar for the two sites, with the highest average monthly temperatures in August in 2020 (25.12 °C and 24.92 °C for C and V, respectively) and July in 2021 (26.24 °C and 25.46 °C for C and V, respectively).

The lower average monthly temperatures were measured in January in both vineyards (in 2020, values of 6.09 °C and 4.98 °C, while in 2021, 5.73 °C and 4.55 °C were recorded for C and V, respectively).

The rainfall recorded in 2020 and 2021 was higher in the Castelplanio vineyard than in the Varano vineyard, except in sporadic cases (January, May, June, and July 2021 and March 2020).

For vineyard C, the wettest months were December (110.8 mm), June (110.6 mm), and May (102.6 mm) in 2020 and November (179.0 mm), December (139.8 mm), and October (138.0 mm) in 2021.

Near the V vineyard, December (92.4 mm), March (84.6 mm), and October (84.2 mm) were the wettest months in 2020, while for 2021, there was greater cumulative rainfall in November (169.4 mm), December (97.0 mm), and January (93.8 mm).

### 2.2. Ecotoxicological Study on Earthworm Eisenia fetida 

#### 2.2.1. Experimental Design

The evaluation of different Cu concentrations' effects on *Eisenia fetida* was carried out using the Varano soil collected in 2020 at a depth of 0–20 cm and belonging to thesis A (Copper treatments: VAZ); the mean total Cu content was 60 mg/kg. To test other concentrations, this soil was further contaminated with the commercial product Siaram 20 WG (Isagro, Milano, Italy), containing copper sulphate. Specifically, fresh solutions of Siaram 20 WG, were prepared and added to the starting soil (VAZ) to reach the final total copper concentrations of 90 mg/kg (VAZ90) and 150 mg/kg (VAZ150). Before starting the ecotoxicological test, all three treatments were incubated for 2 days for equilibration.

In the laboratory, *E. fetida* earthworms were reared at 20 ± 1 °C in organic compost and fed with organic vegetables.

Each treatment was conducted in three replicates consisting of 600 g of dry soil, 12 purged earthworms, and 27% soil moisture.

Five sampling times (2, 7, 14, 21, and 28 days from contamination) were established to follow the evolution of morphological and behavioural changes in the earthworms. All earthworms per replicate were carefully removed from the substrate and observed at each sampling time. Then, one random earthworm per replicate (three earthworms per treatment) was taken for the Comet assay, while the others were returned to the respective vessel.

Any visible change in morphology (narrowing, miniaturisation, lesions, etc.), behaviour (spasms, inability to dig, etc.), and mortality (calculated as percentage variation compared to the initial number of earthworms) were recorded following the OECD guidelines [45,46]. 

#### 2.2.2. Comet Assay

Earthworm coelomocytes were collected following the Eyambe protocol [47] with slight modifications. Briefly, each earthworm was immersed for 4 min at room temperature in an extrusion buffer of 5% ethanol, 95% PBS, 2.5 mg/mL EDTA, and 10 mg/mL guaiacol glyceryl ether (pH 7.3). Coelomocytes were washed and collected by centrifugation (300× *g*, 10 min, 4 °C). The washed cells were counted, resuspended in Low melting agarose (LMA 1%) at 37 °C, and stratified on HT Trevigen slides pre-coated with 1% Normal Melting Agarose (NMA 1%). Each spot was produced by layering LMA containing 3000 cells; each sample was stratified in triplicate. The solidification, lysis, and unwinding phases were carried out following Mincarelli et al. [48]. Electrophoresis was conducted at 11 V/cm for 20 min in a refrigerated room at 4 °C. Slides were washed in H_2_O, neutralised in buffer (0.4 M Tris-HCl buffer adjusted to pH 7.5), and finally dehydrated in 75% methanol [48,49]. Once dried, slides were stained with Sybr Gold, and images were automatically collected using a Lionheart FX Automated Microscope (Biotek, Winooski, VT, USA). Observations were performed at a magnification of 200×. A total of 200 Comet images for each treatment at each time point were acquired in triplicate and subsequently image processed using a custom made analytical software able automatically detect comet and calculate the major DNA damage index: Tail length (TL), Tail moment (TM), and Tail intensity (TI) [50,51]. 

### 2.3. Copper Analyses

The soluble Cu extraction was carried out with distilled water (1 g/10 mL) according to the cession-test reported in the UNI EN 12457-Part 2 [52] while the bioavailable fraction was extracted with a solution of Diethylenetriaminepentaacetic acid (DTPA), CaCl_2_ · 2H_2_O (0.01 M), and triethanolamine (0.1 M) at pH 7.3 (1 g/2 mL), following the indications in the Italian Official Gazette n. 248 [43]. For the total Cu extraction, the protocol in aqua regia by Kasassi et al. [53] was employed, with some modifications: 0.5 g of dry soil and 2 mL of hydrogen peroxide (H_2_O_2_) at 30% were left overnight; after 12 h, the acid attack was carried out by adding 7 mL of HNO_3_. The test tubes were then placed with a float in a preheated water bath (>85 °C) for 15 h; at the end of 15 h, the samples were filtered. According to EPA 6010D [54], the Cu analysis was carried out by inductively coupled plasma optical emission spectrometry (ICP-OES, Agilent mod. 5800).

In the monitoring study, for each of the 12 groups (3 theses × 2 vineyards × 2 depths), the measurements were carried out in five replicates, each corresponding to five sampling points. However, the copper measurements were carried out in three replicates for each treatment in the ecotoxicological study.

### 2.4. Statistical Analysis

Differences in copper concentrations between groups were assessed using a non-parametric test because of the non-normal distributions of the data. Statistical analyses were performed in R software version 4.1.3 [55].

Specifically, the Kruskal–Wallis test was employed to evaluate the presence of significant differences. When this test reported such presence, in order to determine which groups differed from the others, a post-hoc pairwise Dunn test was conducted [56]. A compact letter display was constructed using the cld function of the rcompanion package of R [57] to show significant differences (α = 0.05) between the least-squares means. Groups not sharing any letters are significantly different.

Statistical analysis of Tail intensity data comes from the comet assay performed using the GraphPad Prism version 5 software. The Kolmogov–Smirnov test has been used to check data distribution, and significant differences among groups have been verified using the ANOVA one-way test and Dunn’s post-hoc test.

## 3. Results

### 3.1. Monitoring Study

The sampling campaigns conducted during the two years allowed us to perform an accurate analysis of Cu concentrations in the two soil layers of the experimental vineyards.

**Figure 3:**
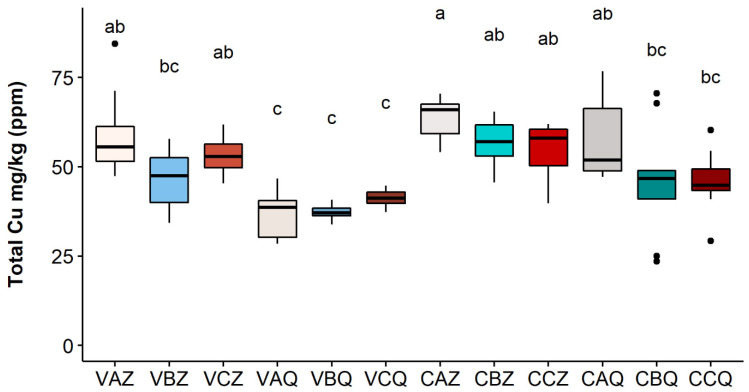
Total Cu concentration in each sample in 2020. Lower case letters refer to Dunn’s multiple comparisons (Benjamini–Hochberg *p*-value adjustment, α-level = 0.05). Code legend: the first letter refers to the vineyard; (C) Castelplanio, (V) Varano. The second letter refers to the thesis: (A) Cu treatments, (B) Alternate treatments with Cu and chitosan, and (C) Chitosan treatments. The third letter refers to the depth: (Z) 0–20 cm, (Q) 20–40 cm.

**Figure 4:**
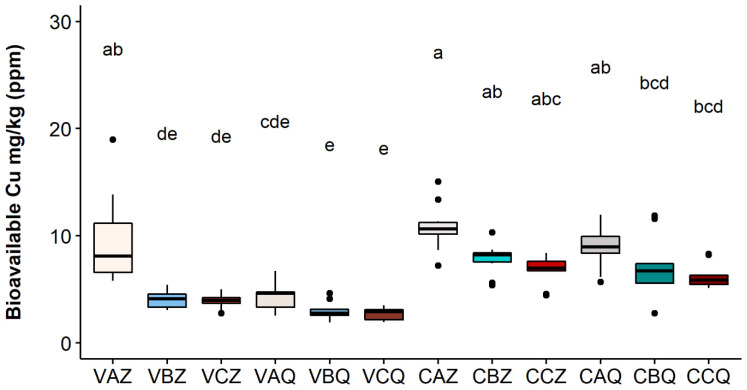
Bioavailable Cu concentration in each sample in 2020. Lower case letters refer to Dunn’s multiple comparisons (Benjamini–Hochberg *p*-value adjustment, α-level = 0.05). Code legend: the first letter refers to the vineyard; (C) Castelplanio, (V) Varano. The second letter refers to the thesis: (A) Cu treatments, (B) Alternate treatments with Cu and chitosan, and (C) Chitosan treatments. The third letter refers to the depth: (Z) 0–20 cm, (Q) 20–40 cm.

**Figure 5:**
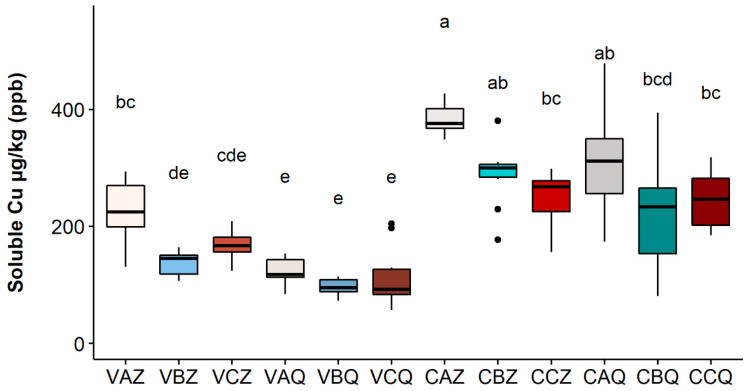
Soluble Cu concentration in each sample in 2020. Lower case letters refer to Dunn’s multiple comparisons (Benjamini–Hochberg *p*-value adjustment, α-level = 0.05). Code legend: the first letter refers to the vineyard; (C) Castelplanio, (V) Varano. The second letter refers to the thesis: (A) Cu treatments, (B) Alternate treatments with Cu and chitosan, and (C) Chitosan treatments. The third letter refers to the depth: (Z) 0–20 cm, (Q) 20–40 cm.

The results for the year 2020 at the two depths of 0–20 and 20–40 cm are reported in Figure 3, Figure 4 and Figure 5, respectively, for the three Cu fractions; total, bioavailable, and soluble.

As shown in Figure 3, the metal is mainly accumulated in the first 20 cm of soil; differences between the two depths are significant in all three theses for the Varano vineyard, while for the Castelplanio vineyard, the trend was confirmed but with no statistically significant differences. It does not seem that the different theses (A, B, and C) have led to significant differences in the concentrations of total copper; however, higher values were found in the thesis in which the Cu dose distributed was the highest (A).

The values of bioavailable copper ranged between 6.59% and 16.96%, with respect to total Cu, among vineyards, theses, and depth. The Varano vineyard, thesis A, where the greatest amount (2.5 kg/ha) of copper was added to the soil, showed a significantly higher concentration of bioavailable Cu than the other two theses in the first 20 cm of soil. The same trend was also found in the Castelplanio vineyard but with values not statistically different.

The values of soluble copper in Figure 5 (in µg/kg) result in very low percentages, compared to the other fractions, with a greater amount of the metal in thesis A in both vineyards.

**Figure 6:**
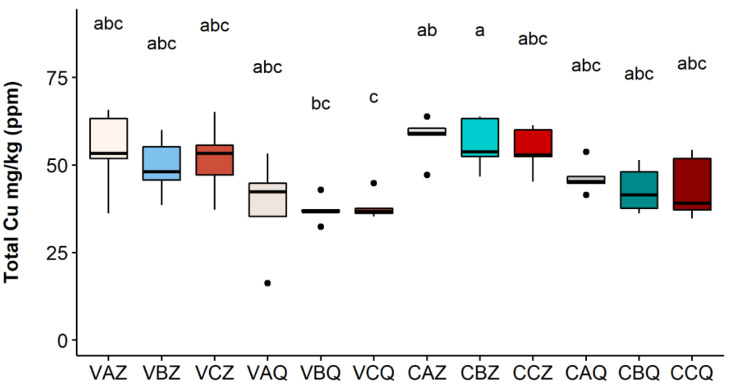
Total Cu concentration in each sample in 2021. Lower case letters refer to Dunn’s multiple comparisons (Benjamini–Hochberg *p*-value adjustment, α-level = 0.05). Codes legend: the first letter refers to the vineyard; (C) Castelplanio, (V) Varano. The second letter refers to the thesis: (A) Cu treatments, (B) Alternate treatments with Cu and chitosan, and (C) Chitosan treatments. The third letter refers to the depth: (Z) 0–20 cm, (Q) 20–40 cm.

**Figure 7:**
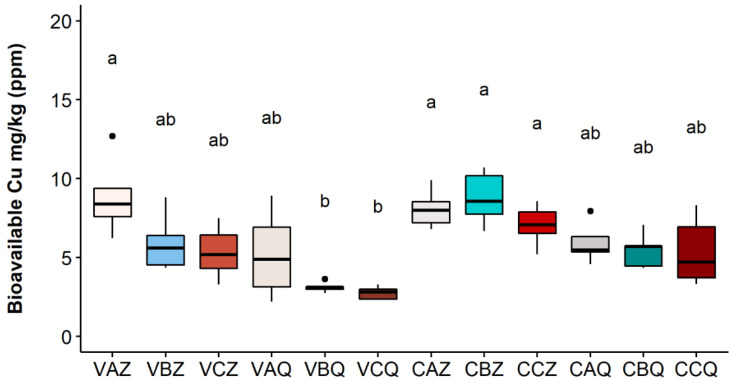
Bioavailable Cu concentration in each sample in 2021. Lower case letters refer to Dunn’s multiple comparisons (Benjamini–Hochberg *p*-value adjustment, α-level = 0.05). Codes legend: the first letter refers to the vineyard; (C) Castelplanio, (V) Varano. The second letter refers to the thesis: (A) Cu treatments, (B) Alternate treatments with Cu and chitosan, and (C) Chitosan treatments. The third letter refers to the depth: (Z) 0–20 cm, (Q) 20–40 cm.

**Figure 8:**
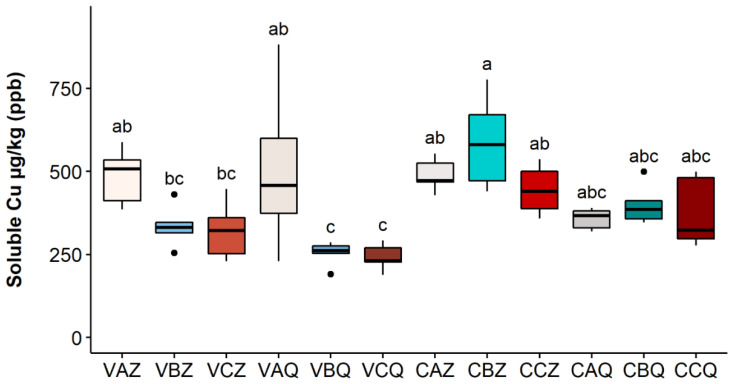
Soluble Cu concentration in each sample in 2021. Lower case letters refer to Dunn’s multiple comparisons (Benjamini–Hochberg *p*-value adjustment, α-level = 0.05). Codes legend: the first letter refers to the vineyard: (C) Castelplanio, (V) Varano. The second letter refers to the thesis, (A) Cu treatments, (B) Alternate treatments with Cu and chitosan, and (C) Chitosan treatments. The third letter refers to the depth: (Z) 0–20 cm, (Q) 20–40 cm.

In analogy with the data reported for 2020, the data relative to the 2021 monitoring campaign are reported in Figure 6, Figure 7 and Figure 8, respectively, for total, bioavailable, and soluble Cu. Even if the trend for the three Cu species in the three theses A, B, and C is similar to the year 2020, no significant differences were found between the three theses. A decreasing trend was observed between the two depths in both vineyards, even if not statistically significant, indicating lower mobility of copper in this second year of experimentation.

Table 3 shows the two-year variations of the three copper fractions for each sample. The variation of Cu concentration was calculated as a percentage of the initial concentration measured in the 2020 monitoring campaign.

In the case of total Cu, a generalised decrease in Cu concentration was observed, but only in the case of the greater depth of the Castelplanio vineyard, thesis with the Cu-treatment (CAQ); the variation between 2020 and 2021 is statistically significant (*p*-value: 0.0199). 

Regarding bioavailable Cu, significant changes were recorded in two samples, VBZ and CAQ (*p*-value: 0.0274 and 0.0181). 

On the contrary, the growth trend of soluble copper is evident and almost always statistically significant (CBQ and CCQ with a *p*-value of 0.0143 and 0.0142; all the other samples, except for CAQ, have a *p*-value < 0.01). However, the changes in concentration during the two years are always referred to as a very little percentage of the total.

### 3.2. Ecotoxicological Study on Earthworm Eisenia fetida

The Cu concentration results within the three treatments were stable during the 28 days of the test and the fluctuations in concentrations are not considered to be significant between the beginning and the end of the test. Consistently, with the fortifications during the entire trial, we see significant differences between the concentrations of the three highest Cu fractions (soluble, bioavailable, and total) measured in the VAZ150 samples as compared to the smaller ones in VAZ (Table S1).

**Figure 9:**
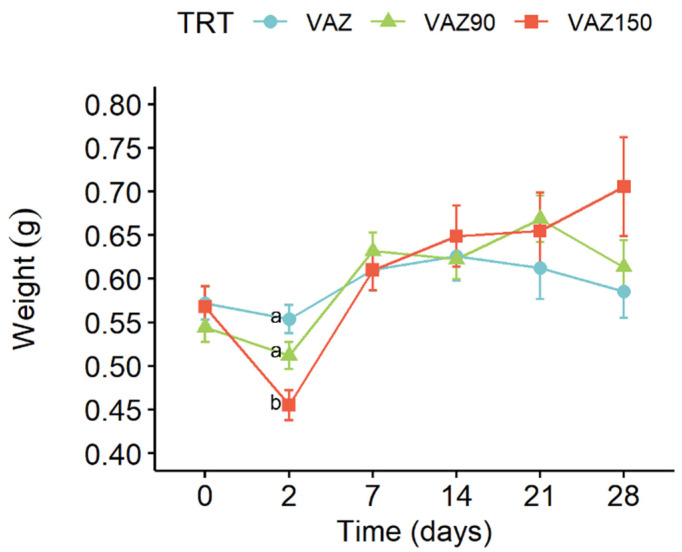
Earthworm's mean weight trend during the ecotoxicology test. According to Dunn’s multiple comparisons (Benjamini–Hochberg *p*-value adjustment, α-level = 0.05), different letters at the same time indicate significant differences between the treatments. When no letter is shown, there are no significant differences between treatments.

As shown in Figure 9, a decrease in mean weight was measured in all three concentrations on day 2, most markedly for the 150 mg/kg dose (VAZ150). The weights at the field concentration (VAZ) and 90 mg/kg (VAZ90), after recovery on day 7, remained almost constant, while the weights of VAZ150 had a recovery trend higher than the other two doses on day 7, and continued to increase until day 28.

The observations conducted on earthworms during the test seem to follow a dose-response trend. The first appearance of morphological anomalies is recorded on day 14 only at the copper dose of 150 mg/kg, while in the case of 90 mg/kg, it was observed from day 21. Unusual behaviour such as lying motionless on the surface and thus inability to dig was found at the end of the test (day 28) at the two fortified doses (VAZ90 and VAZ150). 

Suffering observations were completely absent at the field dose (VAZ) for the entire test period. Mortality was revealed in VAZ150 from day 14 (2.8%), and it increased until the end with a percentage of 5.6 and 8.3, respectively, at 21 and 28 days. 

**Figure 10:**
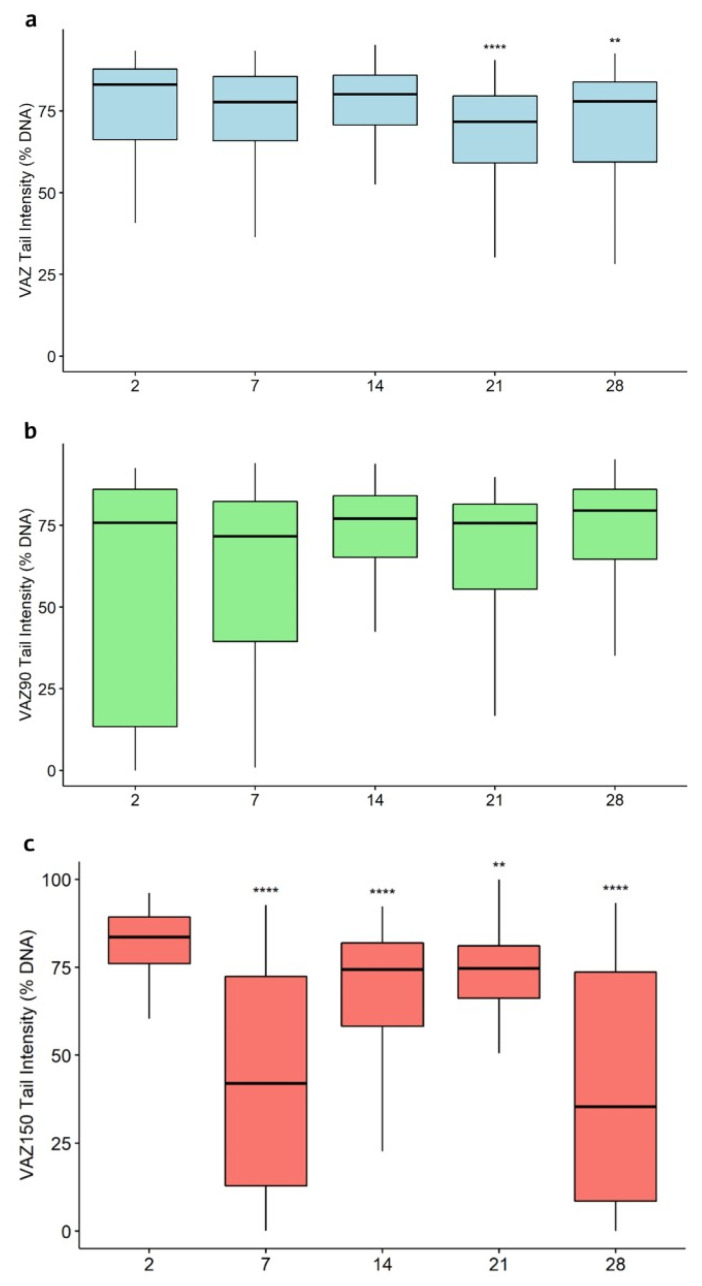
Tail Intensity in earthworms coelomocytes. (**a**) Trial at the field copper concentration (VAZ), (**b**) trial at the fortified copper concentration of 90 mg/kg (VAZ90), and (**c**) trial at the fortified copper concentration of 150 mg/kg (VAZ150). According to Dunn’s test, asterisks relate to significant differences of each time compared to day 2 from contamination (** *p* < 0.01; **** *p* < 0.0001).

Regarding the DNA damage of earthworms, the results in terms of Tail Intensity (TI) are shown in Figure 10.

At the field concentration (VAZ), the DNA damage showed a decreasing trend over time with respect to the damage on day 2 (TI values of 83%), reaching TI values significantly different from 72% and 78% on days 21 and 28, respectively. In the VAZ90 treatment, no significant differences were found between the damage on days 7, 14, 21, and 28 and the initial damage on day 2. 

An evident and significant trend of decrease in DNA damage indexes was observed in the thesis with the highest Cu concentration (VAZ150); indeed, despite the fluctuation measured at 14 and 21 days, the DNA damage always remained significantly lower with respect to that observed on day 2.

Table 4 reports the median Tail Intensity values and the differences in DNA damage at the five sampling times between the test at field Cu concentration (VAZ) and those at 90 and 150 mg/kg (VAZ90 and VAZ150). The comparison showed that at the higher dose of copper, from 7 days onwards, significantly lower DNA damage indexes were recorded except for day 21. On the contrary, the damage levels measured in VAZ90 are similar to those measured in the coelomocytes analysed in the earthworms maintained at the field dose (VAZ).

## 4. Discussion

The monitoring campaign conducted in the years 2020 and 2021, showed a concentration of copper in the first 20 cm of soil higher than that measured in the 20–40 cm at all sampling points. The accumulation of copper in vineyard soil, especially in the first 15 cm of depth, is widespread [58].

This is quite expected, considering that the first layers of soil are those most exposed to the residues of the spraying of copper-based fungicides and that they are also the richest in organic matter (Table 2), which is mainly responsible for the adsorption of Cu [59].

The values of total copper measured ranged between 36.64 mg/kg and 63.82 mg/kg, among vineyards, theses, and depth, showing that the cupric treatments carried out over the years of cultivation have scrupulously followed the European and national laws regulating the Cu amount to be used in organic farming setting at 4 kg/ha/year [60]. Values found are lower for both depths than the contamination threshold concentration for agricultural areas set at 200 mg/kg by the Italian Decree n° 46 [61], and still, they are always below 100 mg/kg, which is the most stringent limit in Italy for the copper content in agricultural soils destined for fertilisation with sewage sludge [62].

At the European level, there is a lot of variability between nations regarding the limits of metals in agricultural soils. For example, the values indicated in Germany, Belgium, the Czech Republic [63], and Poland [64] which provide thresholds of 20–60, 50–72, 100, and 150 mg/kg of copper, are lower than the Italian ones. On the other hand, some nations indicate higher levels of copper, i.e., Denmark [65] and Portugal [66], with 500 and 1000 mg/kg, respectively. At a European level, therefore, there is no univocal standard regarding the concentrations of copper allowed in agricultural soils. Still, there is a guideline relating to agricultural soils in which sewage sludge is applied, establishing reference concentrations for some metals. For copper, these values are in the range of 50 to 140 mg/kg and can be considered a reference range for environmental risk [67,68].

The concentrations found in the present work and the high variability of the data obtained are similar or lower if compared with the copper accumulation values in vineyard soils found in reports of various Italian Regions [19,20]. The mean Cu concentration measured by the LUCAS topsoil survey in 25 European nations was 16.86 mg/kg, with high variability; vineyards are the land cover class with the highest percentage of soil samples displaying higher than 100 mg/kg [8].

From the ecotoxicological point of view, it is important to determine the bioavailable Cu because it represents the fraction of the total metal content in the soil that biota can utilise [21]. The values of bioavailable copper as a percentage of the total Cu obtained in this monitoring are certainly lower than the average value of 32% found in the measurement campaign carried out by the Region Veneto, Italy, in 2019 [69].

Observing the values of soluble copper, always in the order of ppb, are a minimum percentage if compared to the other forms. Therefore, the research suggests that the risks of copper diffusion by leaching towards the deep layers, flowing towards surface waters, or erosive transport with meteoric waters are minimal in the conditions tested.

The high pH values of these soils can also contribute to insolubilise Cu in the form of hydroxide [70], whereby the bioavailable and soluble forms can be limited to very lower percentages of the total than other soils, with values ranging from 7.24% to 16.96% of bioavailable copper and from 0.26% to 1.33% of soluble copper, with respect to the total copper. Finally, organic cultivation allows the soils to retain metals much more than the soils conventionally cultivated and often transforms them into insoluble or unavailable chemical species [71]. The characteristics of high CSC, pH, and organic substance of the tested soils favour the metal’s immobilisation and reduce its absorption by plants and microorganisms, preventing them from toxic level uptakes as reported elsewhere [72]. The reduction in the availability of copper can positively influence bacterial biodiversity, which has been proven to be sensitive to this metal in the soil [73]. On the other hand, in terms of edaphic biota, it seems that fungal richness is resilient to copper, in fact, according to Keiblinger et al., the effects on the number of taxa are transitory [74].

The changes in the total copper concentration between the two years under study were not significant, and in any case, they decreased in most of the samples. This could be considered a positive aspect from an environmental point of view, though further investigation on the fate of Cu, including plant uptake, leaching in the deeper layers, and runoff and erosion are necessary. The high number of no significant changes in bioavailable Cu concentration, joined with similar behaviour of the total Cu species, allow us to affirm that the management of Cu treatments in the two vineyards tested can be considered appropriate and does not represent a significant impact from an environmental point of view. 

Only the soluble copper significantly increased in both vineyards in 2021. The temperatures observed at the two sampling sites followed a very similar trend, having higher monthly average summer temperatures in 2021 compared to 2020. In terms of rainfall, the Castelplanio vineyard received higher water supplies than Varano, both in 2020 and 2021, which may have influenced the greater leaching of copper in the Castelplanio vineyard compared to that of Varano; the soluble copper increase was less evident (range of +13.80% to +50.97%) than the percentage increase recorded in the samples of Varano (range of +48.26% to +76.05%). In any case, it should be borne in mind that we are referring to very low concentrations with respect to the total Cu, thus, it is challenging to establish the climatic impact on the behaviour of this fraction in the soil.

Regarding the ecotoxicological study conducted with earthworms, in all treatments (VAZ, VAZ90, and VAZ150), the three copper fractions in the soil did not change significantly over time. The treatments at field concentration (60 mg/kg) and 90 mg/kg seemed to generate the same effect in terms of the values of the three metal fractions over time and were not associated with any sign of harm to earthworms such as abnormal behaviour or death. Comet assay results confirmed what had been observed in the weight trend: it seems that in the case of the higher dose (VAZ150), an acute insult was observed in the first 2 days of exposure followed by a recovery in the subsequent days, with an increase in the average weight correlated to the noticeable decrease in DNA damage. Duan et al. [72] found weight gain in earthworms subjected to sub-lethal copper concentrations with ranges similar to those tested in the present study (below 320 mg/kg) and assumed that biomass and survival alone are not parameters that give a complete view of toxic effects.

The results found in the present study are in agreement with those described in a review by La Torre et al. [75] on Cu use in agriculture; even though earthworms may avoid toxic Cu levels by migrating to uncontaminated soil, they may also adapt to certain levels of contamination with the development of biochemical mechanisms of detoxification [22,31]. 

Some authors confirm the concepts of adaptation to copper doses comparable with the highest amount present in this experiment; Mincarelli et al. [35] found that nine days after the treatment, TI was significantly lower than in the previous days; authors ascribed this behaviour to the increasing level of metallothioneins, at a dose of 120 mg/kg, which are responsible for copper detoxification. 

Nonetheless, mortality of 8.3% was recorded at the end of the experiment in the treatment with 150 mg/kg of copper, suggesting a toxic effect of Cu on earthworms in these experimental conditions. 

## 5. Conclusions

The monitoring campaigns in the two years of experimentation highlight that the vineyard management regarding Cu-treatments in the two farms involved in the investigation was prudent. The concentrations of the three forms of copper did not undergo significant variations. In the two-year monitoring, an overall decrease in total Cu concentration in soil occurred, and this can be considered a positive factor from an environmental point of view. However, frequent leaching and/or runoff events could be associated with increases in the concentration of Cu in surface and deep waters, which could cause concern. Particular attention should be paid to the types of copper-based fungicides used, possibly preferring slow-release ones to avoid environmental dispersion as much as possible. 

In the ecotoxicological experiment, the highest dose of 150 mg/kg caused an initial toxic effect, higher than the other two doses, that was followed by adaptive responses in the earthworms that lead to DNA repair and overall weight increases. It can be stated that at the field concentration (60 Cu mg/kg), the ecotoxicological effects on non-target species worm *Eisenia fetida* are negligible. Even at artificially increased doses of 90 and 150 mg/kg, the adverse effects regarding earthworm weight and DNA damage are transient and not worrying. Further research is needed on the ecotoxicological effects of Cu on soil nontarget organisms, by testing a wider range of copper concentrations and involving a novel type of biomarker to analyse.

## Figures and Tables

**Table 1:** Summary of the number of Cu-treatments carried out during the last two years in the vineyards under study.

Vineyards	Thesis	N° of Cu Treatments/Year	Year
Varano (V)	A	9	2020
		7	2021
	B	4	2020
		5	2021
Castelplanio (C)	A	9	2020
		5	2021
	B	4	2020
		4	2021

**Table 2:** Summary of main physico-chemical characteristics of the soils under experiment, analysis protocols by Italian Official Gazzette n° 248 [43].

Sample Code ^1^	pH (H_2_O)	Texture	Sand	Silt	Clay	Conductivity	OM	CEC
			%			dS/m	%	Meq/100 g
**VAZ**	8.1	Silty Clay Loam	19.5	51.0	29.5	0.644	2.53	24.5
**VAQ**	8.2	Clay Loam	20.8	49.0	30.2	0.587	1.26	25.6
**VBZ**	8.2		21.5	45.6	32.9	0.61	2.05	30.9
**VBQ**	8.2		20.4	47.3	32.3	0.536	1.50	30.6
**VCZ**	8.2	Silty Clay Loam	19.9	48.5	31.6	0.627	2.02	29.1
**VCQ**	8.2		15.4	50.6	34.0	0.516	1.69	29.6
**CAZ**	8.1	Loam	44.3	29.8	25.9	0.638	2.22	25.4
**CAQ**	8.2	Sandy Clay Loam	47.6	27.1	25.3	0.597	1.18	24.5
**CBZ**	8.1	Loam	41.0	33.6	25.4	0.869	2.28	22.4
**CBQ**	8.2		42.4	31.5	26.1	0.731	1.33	22.0
**CCZ**	8.1		43.3	31.5	25.2	0.614	2.02	24.1
**CCQ**	8.1		41.3	33.4	25.3	0.717	1.38	21.9

^1^ Codes legend: the first letter refers to the vineyard: (C) Castelplanio, (V) Varano. The second letter refers to the thesis: (A) Cu treatments, (B) Alternate treatments with Cu and chitosan, and (C) Chitosan treatments. The third letter refers to the depth: (Z) 0–20 cm, (Q) 20–40 cm.

**Table 3:** Percent of variation in copper concentration in the two years of experimentation.

Sample Codes ^1^	Cu Percentage Variation between Two Years (%)		
	Tot	Bio	Sol
VAZ	−8.64	−8.61	+53.27 **
VBZ	+5.68	+32.15 *	+59.12 **
VCZ	−3.07	+26.71	+48.24 **
VAQ	+4.54	+15.59	+76.05 **
VBQ	−0.14	+4.87	+59.12 **
VCQ	−8.59	+1.19	+52.66 **
CAZ	−10.40	−33.75	+21.31 **
CBZ	−0.66	+10.40	+50.97 **
CCZ	−0.26	+3.22	+44.13 **
CAQ	−24.44 *	−51.51 *	+13.80
CBQ	−7.11	−25.03	+43.73 *
CCQ	−5.47	−15.04	+34.56 *

Significance of Cu variation between 2020 and 2021 was calculated in each sample according to Dunn’s test (* *p* < 0.05; ** *p* < 0.01; Benjamini–Hochberg *p*-value adjustment, α-level = 0.05). Abbreviation Legend: Tot refers to total Cu, Bio refers to bioavailable Cu, and Sol refers to soluble Cu. ^1^ Code legend: the first letter refers to the vineyard; (C) Castelplanio, (V) Varano. The second letter refers to the thesis: (A) Cu treatments, (B) Alternate treatments with Cu and chitosan, and (C) Chitosan treatments. The third letter refers to the depth: (Z) 0–20 cm, (Q) 20–40 cm. + indicates an increase in Cu concentration, - indicates a decrease in Cu concentration.

**Table 4:** Tail intensity (TI) median values during the ecotoxicological study.

Treatments	Time (Days)				
	2	7	14	21	28
VAZ	83%	78%	80%	72%	78%
VAZ90	78% *	72% *	77%	76%	80%
VAZ150	83%	42% ****	74% ***	75%	35% ****

According to Dunn’s test (* *p* < 0.05; *** *p* < 0.001; **** *p* < 0.0001), asterisks relate to significant differences in each time between fortified treatments (VAZ90 and VAZ150) and the field concentration (VAZ).

## Data Availability

Data is contained within the article.

## Supplementary Materials

The following supporting information can be downloaded at: https://www.mdpi.com/article/10.3390/toxics10060310/s1, Table S1: Copper concentrations (mg/kg) during the ecotoxicological test.
