# Supplementary material for: Copper Monitoring in Vineyard Soils of Central Italy Subjected to Three Antifungal Treatments, and Effects of Sub-Lethal Copper Doses on the Earthworm Eisenia fetida
Source: Toxics. 2022 Jun 8;10(6):310. doi: 10.3390/toxics10060310 (PMC9228243; doi:10.3390/toxics10060310)
Supplement: Supplementary file 1 [file toxics-10-00310-s001.zip › toxics-1684269-supplementary.pdf]

toxics

MDPI

Supplementary Materials: Copper Monitoring in Vineyard Soils of Central Italy Subjected to Three Antifungal Treatments, and Effects of Sub-Lethal Copper Doses on the Earthworm *Eisenia fetida*

Arianna De Bernardi, Enrica Marini, Cristiano Casucci, Luca Tiano, Fabio Marcheggiani and Costantino Vischetti

Table S1. Copper concentrations (mg/kg) during the ecotoxicological test.

Time (days)	VAZ			VAZ90			VAZ150		
	Tot	Bio	Sol	Tot	Bio	Sol	Tot	Bio	Sol
2	62.30 ± 1.71	9.64^{b} ± 0.13	0.49^{ab} ± 0.03	104.80 ± 9.19	25.33^{ab} ± 0.60	0.60 ± 0.01	*136.10 ± 0.20	*55.08 ± 0.71	$*0.70^{b}$ ± 0.03
7	-	13.00^{ab} ± 0.24	0.50^{ab} ± 0.03	-	23.92^{ab} ± 1.03	1.08 ± 0.12	-	*53.10 ± 2.83	1.02^{ab} ± 0.12
14	59.50 ± 0.53	13.78^{a} ± 0.10	0.58^{a} ± 0.04	87.73 ± 1.61	26.60^{a} ± 0.57	0.77 ± 0.03	*138.70 ± 7.77	*43.38 ± 1.42	$*1.22^{a}$ ± 0.09
21	-	13.90^{a} ± 0.37	0.57^{ab} ± 0.06	-	17.37^{b} ± 0.89	0.60 ± 0.39	-	*49.65 ± 4.93	1.08^{ab} ± 0.03
28	60.23 ± 0.22	11.95^{ab} ± 1.34	0.45^{b} ± 0.01	96.88 ± 2.47	19.73^{b} ± 3.59	0.60 ± 0.03	*134.78 ± 3.29	*35.93 ± 5.83	$*1.01^{ab}$ ± 0.07

Different letters in the same column (same treatment) indicate significant differences between the times; Asterisks on the same line (same time of sampling) indicate significant differences of fortified treatments (VAZ90 and VAZ150) respect the baseline soil (VAZ). Dunn's test for multiple comparisons following the Kruskall-wallis test significant result, Benjamini–Hochberg p-value adjustment, α-level = 0.05; Abbreviations Legend:Tot refer to total Cu, Bio refer to bioavailable Cu and Sol refer to soluble Cu.
